# A Perspective from a Case Conference on Comparing the Diagnostic Process: Human Diagnostic Thinking vs. Artificial Intelligence (AI) Decision Support Tools

**DOI:** 10.3390/ijerph17176110

**Published:** 2020-08-22

**Authors:** Taku Harada, Taro Shimizu, Yuki Kaji, Yasuhiro Suyama, Tomohiro Matsumoto, Chintaro Kosaka, Hidefumi Shimizu, Takatoshi Nei, Satoshi Watanuki

**Affiliations:** 1 Department of General Medicine, Showa University Koto Toyosu Hospital, Tokyo 135-8577, Japan; haradataku@med.showa-u.ac.jp; 2 Department of Diagnostic and Generalist Medicine, Dokkyo Medical University Hospital, Tochigi 321-0293, Japan; 3 Department of Internal Medicine, Itabashi Chuo Medical Center, Tokyo 174-0051, Japan; ykaji@iuhw.ac.jp (Y.K.); sougoushinryouka@ims.gr.jp (C.K.); 4 Division of Rheumatology, JR Tokyo Hospital, Tokyo 151-8528, Japan; y-suyama@jreast.co.jp; 5 Department of General Medicine, Nerima Hikarigaoka Hospital, Tokyo 179-0072, Japan; tomohiroma@jadecom.jp; 6 Department of Respiratory Medicine, JCHO Tokyo Shinjuku Medical Center, Tokyo 162-8543, Japan; shimizu-hidefumi@shinjuku.jcho.go.jp; 7 Department of Infection Control and Prevention, Nippon Medical School Hospital, Tokyo 113-8602, Japan; takahitonei@gmail.com; 8 Division of Emergency and General Medicine, Tokyo Metropolitan Tama Medical Center, Tokyo 183-8524, Japan; satoshi_watanuki@tmhp.jp

**Keywords:** artificial intelligence, decision support tool, diagnostic process

## Abstract

Artificial intelligence (AI) has made great contributions to the healthcare industry. However, its effect on medical diagnosis has not been well explored. Here, we examined a trial comparing the thinking process between a computer and a master in diagnosis at a clinical conference in Japan, with a focus on general diagnosis. Consequently, not only was AI unable to exhibit its thinking process, it also failed to include the final diagnosis. The following issues were highlighted: (1) input information to AI could not be weighted in order of importance for diagnosis; (2) AI could not deal with comorbidities (see Hickam’s dictum); (3) AI was unable to consider the timeline of the illness (depending on the tool); (4) AI was unable to consider patient context; (5) AI could not obtain input information by themselves. This comparison of the thinking process uncovered a future perspective on the use of diagnostic support tools.

## Perspective

Artificial intelligence (AI) has greatly contributed to the healthcare industry. Specialists have reviewed the relevance of AI in various fields within medicine in many journals [1]. By processing vast amounts of data, AI can predict patterns that cannot be deciphered by biostatistics, and can improve the reliability of predictive models by correcting algorithmic errors. AI has already been applied to image analysis in radiology, pathology, and dermatology, where its diagnostic speed has surpassed that of experts, and its accuracy is now on par with experts. While the diagnostic accuracy of AI will never reach 100%, diagnostic performance may improve when AI is combined with the diagnostic capability of physicians [2]. Besides, in the case of rare diseases, AI has been reported to propose a diagnosis that is earlier and more accurate than a clinical diagnosis [3]. However, physicians have also been reported to have better diagnostic accuracy than computers [4]. Thus, while in some domains AI can serve to complement human capabilities, at present AI does not completely surpass human diagnostic capabilities. All things considered, the best approach would thus be using AI to assist, rather than to replace, physicians in diagnosing patients. However, the effect of AI on diagnosis is not well explored and remains a controversial area [5]. Similar to how shogi, go, and chess are played between a computer and a human player, here we report the advantages and disadvantages of using a decision support tool based on data from a clinical conference in Japan and the literature, through a trial comparing the thinking process between a computer (Isabel©, Ann Arbor, MI, USA) and a master in diagnosis.

The Tokyo General Internal Medicine (GIM) Conference is a joint conference of various teaching hospitals focused on clinical diagnosis and is held every month, mainly in Tokyo, with the aim of providing learning opportunities in diagnostic reasoning [6]. At this conference, differences in diagnostic processes and the actual diagnostic outcomes between a human physician and AI are evaluated. The human diagnostician for this event was a physician designated by the educational committee of the conference (Taro Shimizu, MD, Professor, Chair and Chief of the Department of Diagnostic and Generalist Medicine, Dokkyo Medical University, Tochigi, Japan).

In the conference, Isabel© was used as a decision support tool. Among the many decision support tools currently available, Isabel© has been identified as one that provides excellent outcomes, with diagnoses taking approximately only 1 min per patient and resulting in an improved diagnostic accuracy and a reduced error rate [7,8]. Studies comparing decision support tools have shown Isabel©, Ann Arbor, MI, USA and DxPlain^®^, Boston, MA, USA as superior tools among the others [7]. Although Isabel© is not very good at guessing the first candidate for a differential diagnosis, it has been reported to be the most comprehensive tool in the differential diagnosis of the top 20 candidates [9]. In general practice, physicians have better diagnostic accuracy than AI, but for rare diseases, AI performs better than humans [3,9]. Recent advances in AI, such as deep learning in Alpha-Go, have been tremendous, and clinicians will have to make better use of decision support tools and AI in the future, which is the reason why we held the conference.

During the event, an unknown diagnosis case was presented by a teaching hospital selected by the conference committee. The diagnostician used a whiteboard to organize and exhibit his clinical reasoning strategy. The AI, on the other hand, generated a list of differential diagnoses based on the keywords that were entered. The two methods were compared, as well as the different diagnostic processes that led to the final differential diagnosis. The keywords for AI were selected by 80 physicians participating in the conference. Most of the participants were general physicians with extensive experiences in difficult-to-diagnose cases.

The case evaluated at the conference was that of a 71-year-old man with a history of pulmonary tuberculosis, chronic obstructive pulmonary disease (COPD), and pneumothorax, who was admitted to the hospital after arriving at the emergency department with symptoms of respiratory discomfort for four weeks and vomiting and appetite loss for two weeks. Acute exacerbation of COPD was suspected, and the patient was treated with short-acting β-agonist inhalation, prednisone, and ceftriaxone, which resulted in an alleviation of symptoms. The patient was discharged from the hospital after ten days. He did not experience a progression of symptoms during hospitalization while he once had a transient mild headache. After discharge, he had a recurrence of anorexia. He was able to drink fluids but was unable to eat solid food. Other symptoms included diarrhea without abdominal pain, insomnia, and depression. There were no symptoms of fever, black or bloody stools, joint pain, or skin rash. Due to worsening symptoms, the patient was readmitted to the same hospital three weeks after the first admission. The vital signs were as follows: blood pressure 138/90 mmHg, heart rate 112 beats per minute, body temperature 35.9 °C, respiratory rate nine breaths per minute, and SpO_2_ 94% on ambient air. Physical examination revealed xerostomia, dental caries, a barrel thorax, and bilateral coarse crackles in the lung bases. Otherwise, there were no abnormalities in the abdomen or extremities. There was only a slight consolidation of the right upper lung on contrast-enhanced computed tomography of the whole body, but no major abnormalities on laboratory tests, including blood cell counts and basic chemical panels, and upper and lower endoscopy.

The keywords collected from the audience and entered into Isabel© were “respiratory discomfort”, “fatigue”, “nausea”, and “dysphagia.” The main differential diagnoses output by Isabel© based on these keywords included cardiovascular disease, myasthenia gravis, pulmonary tumor, lymphoma, sarcoidosis, and iron-deficiency anemia. Considering the pathological process, the diagnostician formulated differential diagnoses comprehensively based on System 2, by ranking their probabilities according to the patient’s progression and availability of new information.

When determining the history of the present illness based on the patient’s ability to drink water and inability to eat solid food, the physician initially considered esophageal diseases (e.g., achalasia) and intrathoracic space-occupying lesions. However, based on diagnostic imaging, the diagnostician judged that the patient’s condition was highly unlikely to be achalasia or intrathoracic space-occupying lesions. The diagnostician focused on the information that “the symptoms were spread over multiple organs” and “the respiratory rate was 9 breaths per minute”, and recalled the possibility of a metabolic/endocrine disease and central nervous system disorder. Based on the above information, the diagnostician focused on central adrenal insufficiency as the most likely diagnosis, such as in the pituitary gland and hypothalamic region, followed by paraneoplastic syndrome, and atypical Guillain-Barré syndrome, and considered these as the top three differential diagnoses.

Subsequently, a low morning cortisol level (0.7 μg/dL) triggered a close endocrine examination, and a final diagnosis of pituitary dysfunction was made. Although the patient was unable to eat solid foods, no organic abnormalities of the nervous or gastrointestinal systems were found, and dysphagia and diarrhea disappeared after steroid replacement therapy.

From developments in psychology, “Dual-process’’ theories suggest that classification decisions are made either by a fast, unconscious, contextual process called System 1 or by a slow, analytical, conscious and conceptual process called System 2. System 1 thinking, experience-based and automatic, is consistent with memories containing individual experiences and access based on similarity between the present situation and prior experience. By contrast, System 2 thinking is conscious, logical, and contextual, access based on abstract concepts or rules, contain a logical combination of individual features [10]. Initially, the physician set a diagnosis based on intriguing history keywords through the System 1 process. When the initial diagnosis became unlikely, given the imaging results, he calibrated his thinking process and revisited the history, and re-examined the consistency of the constellation of clinical information. He then picked up and weighed unexplained but possibly important information, such as headache and bradypnea, in the context of multiple organ dysfunction, considering the timeline of the presentation, and possible triggers of acute exacerbation of the chronic and comorbid condition. The physician then specified the most likely diagnosis and made differential diagnoses. Meanwhile, AI was unable to demonstrate thought processes, and the differential diagnosis did not include the final diagnosis of endocrine disorders, including pituitary dysfunction. In this case, “being able to drink water and unable to eat solid food” was a characteristic symptom, but one of the points of contention was how to interpret it. There were complaints of dysphagia, but there were no actual gastrointestinal or neurological abnormalities. The differential diagnosis was based on the patient’s symptoms, excluding “dysphagia”, and Isabel© presented endocrine disorders that included adrenal insufficiency. This fact illustrates both the magnificent comprehensiveness of Isabel©’s differential diagnosis and the danger of giving misleading information, depending on the keywords that humans enter.

This discussion regarding the use of decision support tools in the context of the comparison with human diagnostic thinking underscored the following issues: (1) input data (history and physical examination findings) could not be weighted in order of importance for diagnosis; (2) AI could not deal with comorbidities (see Hickam’s dictum); (3) depending on the tool, AI may be unable to consider the timeline of the illness; (4) AI was unable to consider patient contexts such as social background and other information not explained by keywords; and more importantly, (5) AI could not obtain input information by themselves. Input information plays an important role when physicians create a list of differential diagnoses. Moreover, an even bigger challenge was that the lack of clarity in AI’s diagnostic reasoning process made AI fail in terms of patient explanation and education as well as under- and postgraduate medical education, because of the fact that machine learning cannot always display a thinking process that is understandable by humans.

Reliance on decision support tools that do not clearly demonstrate logical thinking processes that humans can understand may have a negative impact on learners. Novices who do not have logical thinking processes may tend to overly depend on decision support, such as AI, and such dependence is associated with negative effects. A systematic review by Goddard et al. also described that overreliance on decision support may reduce clinicians’ independent judgment and critical thinking [11]. Federico et al. also warned that dependence on AI perpetuates “de-skilling”, or attrition in human skills [12]. As human input is required for AI optimization, this “de-skilling” could be a major threat to diagnostic accuracy [5]. There are some other disadvantages of using the diagnostic decision support tool. Because the decision support tool is used by humans, it is difficult to avoid performance degradation caused by the surrounding environment, fatigue, and interruptions. Experienced doctors who use a System 1 thinking process may use a decision support tool to avoid pitfalls, but novice doctors who use a System 2 thinking process may need additional information to use a decision support tool, which may take a longer time. Additionally, incomplete information input and outdated guidelines may lead to erroneous advice [13].

However, in the area of diagnosis, AI has various advantages. First, AI is not affected by fatigue, distraction, or data overload and always has a consistent performance [13]. Second, AI can be used to group patients based on electronic health record information and optimize them for individual institutions [14]. Third, because of the complexity of human physiology and the diversity of human diseases, AI will not make absolute decisions [15]. Experienced clinicians who frequently use a System 1 diagnostic process may use the completeness of the differential diagnosis by AI to avoid pitfalls [13]. Considering these factors, a clinical decision support system can work as an adjunct, improving the clinician’s behavior [16] and diagnostic accuracy [17,18].

The benefits and disadvantages of AI in the diagnostic field are listed in Table 1. Physicians are human beings, and it is impossible for them to completely avoid the effects of fatigue and the surrounding environment on their performance and biases in decision-making. However, only physicians can understand the patient’s psychosocial context, assign weights to information, and translate medical history into medical terms. Meanwhile, AI is a consistent system not affected by fatigue, bias, interruptions, and data overload. However, as AI does not yet have a good understanding of natural language processing, nor can it accommodate the complexity of the human physiology and diversity of human diseases, it can only play an advisory role, rather than making absolute decisions. As an example, for less experienced radiologists or less trained physicians, AI could potentially enhance diagnostic accuracy and therefore contribute to patient safety. However, this is also a double-edged sword because the implementation of AI could lead to physician deskill. AI does not show processes, but results alone, and does not provide the information for improving the beginners’ diagnostic thinking. Hence, AI can have a negative impact on the training of diagnostic skills in beginners, although it is sometimes useful to them in a palliative way. These advantages and disadvantages of AI are complementary, and doctors need to understand them well.

There are four limitations to this article: First, this article is a comparison of the diagnostic process of a single diagnostically challenging case. Therefore, further studies may be needed to secure the external validity of the study result. Second, we need to acknowledge the issue regarding using Isabel as a tool. This study examined only a single AI support tool (i.e., Isabel). There are other AI diagnostic support tools in the market, which might show different diagnostic results. Further studies with these tools will contribute to establish general conclusion in evaluating diagnostic AIs. Third, it has been said to be hard to evaluate the diagnostic accuracy of AI. The output or response from AI is qualitative variables, and traditional accuracy measures such as sensitivity, specificity and ROC are not applicable. Some evaluation measures to evaluate the performance of qualitative response variables, such as the Single model evaluation (Hypervolume under the manifold, Correct classification probability, R-squared value and Polytomous discrimination index) and Model comparison (Net reclassification improvement and Integrated discrimination improvement), have been considered [19], which we look forward to utilizing. In addition, the fourth limitation is the characteristics of the diseases addressed in this case. Deep learning usually requires a large amount of input data. The characteristic of this case had an atypical presentation with uncommon disease. In such a case, the challenge is that the acquisition of input data is scarce, hence obtaining data of uncommon and atypical diagnostic cases may raise an issue in establishing the quality of AI tackling with diagnostically difficult cases.

In summary, diagnostic support tools can be effective when used to augment the diagnostic process. An understanding of the advantages and disadvantages of AI-based diagnostic support tools is likely to increase the comprehensiveness of diagnoses made by physicians and reduce human error and diagnostic errors.

## Figures and Tables

**Table 1:** Benefits and disadvantage of AI.

Benefits	Disadvantage
By processing vast amounts of data, AI can predict patterns and improve the reliability of predictive models	
AI can improve clinician behavior and diagnostic accuracy	AI’s diagnostic accuracy will never reach 100%
Diagnostic accuracy of rare diseases is better than that of physicians	AI’s diagnostic accuracy is inferior to that of physicians
	AI cannot be weighted in order of importance
	AI cannot or does not deal with co-morbidities
	Depending on the tool, AI may be a lack of consideration of the timeline of the illness
AI can be grouped from the EHR information and optimized for individual institutions	AI cannot consider patient contexts such as social background and other information
	AI cannot obtain input information by themselves
	Lack of clarity in the diagnostic reasoning process
	Over-reliance may reduce clinicians’ independent judgment and critical thinking(de-skilling)
AI is not affected by fatigue, distraction, or data overload, and is consistent and always the same Performance.	AI is used by human beings, cannot avoid performance degradation caused by the surrounding environment, fatigue, and interruptions.
Experienced clinicians who frequently use the system1 diagnostic process may use the completeness of the differential diagnosis by AI to avoid pitfall	Novice physicians using the system 2 thought process may need additional information to determine the validity of the AI’s recommendations.
	Incomplete information input and outdated guidelines may lead to erroneous advice
Because of the diversity in human physiology and disease, AI will not make absolute decisions	
